# Food Safety Issues Related to Eating In and Eating Out

**DOI:** 10.3390/microorganisms10112118

**Published:** 2022-10-26

**Authors:** Adriana H. Gargiulo, Stephany G. Duarte, Gabriela Z. Campos, Mariza Landgraf, Bernadette D. G. M. Franco, Uelinton M. Pinto

**Affiliations:** 1 Department of Food and Experimental Nutrition, Faculty of Pharmaceutical Sciences, University of Sao Paulo, Sao Paulo 05508-000, Brazil; 2 Food Research Center, Faculty of Pharmaceutical Sciences, University of Sao Paulo, Sao Paulo 05508-000, Brazil; 3 International Life Sciences Institute Brazil, Sao Paulo 01449-070, Brazil

**Keywords:** eating out, microbiological contamination, food safety, foodborne disease, dark kitchens

## Abstract

Because of growing urbanization and lack of time to prepare meals at home, eating out or getting food delivered have become common trends for many people. The consumption of food from unknown sources may impose an increased chance of contamination with microbiological hazards, especially if sanitary conditions are not met. We evaluated data from health surveillance agencies and scientific articles on foodborne diseases (FBD) reported internationally according to the exposure sites. We observed that the data are influenced by cultural, political, and socioeconomic differences. For instance, in New Zealand, Australia, United States, Denmark and India, the occurrence of FBD outbreaks was greater from foods prepared in commercial establishments and street vendors than from households. Conversely, in China, countries of the European Union and Brazil, the results are the opposite. Additionally, the pandemic imposed new eating behavior patterns, increasing delivery services and foods prepared in so-called “Dark Kitchens”. The underreporting and heterogeneity of data among countries prevented a precise conclusion to the question of whether homemade foods are inherently safer than foods prepared out. Nevertheless, a lower level of development in a country influences its sanitation conditions, as well as the number of street food vendors, the search for cheaper foods, and insufficient knowledge of the population on good hygiene practices, which can all increase the chances of FBD cases.

## 1. Introduction

Food and nutrition are crucial to health and human development. Well-nourished children learn better, and adults become more productive and able to break intergenerational cycles of hunger and poverty [1]. However, food can undergo chemical or microbiological contamination during production, distribution, preparation and/or storage, making it unsafe for human health, which has resulted in alarming annual estimates of 600 million cases and 420,000 deaths and the loss of 33 million years lived in health worldwide due to foodborne diseases (FBD) [2,3]. Despite this significant public health, economic and social burden, and the need for greater awareness, these estimates face substantial data gaps, making it difficult to identify priorities at the national level, in terms of diseases, hazards, locations and foods that most contribute, so that effective interventions for prevention may be adopted. Data on the FBD burden are needed to prioritize food safety policies and allocate resources where most needed. While many countries recognized the need for studies on the national burden of FBD and adopted measures to implement them, particularly those with high incomes, many others still lack public commitment, technical and financial resources to estimate this burden; barriers that can be increased in the face of other post-pandemic demands [4].

In addition to non-biological factors, bacteria and their toxins, viruses and other parasites can cause more than 250 types of FBD [3,5,6]. Non-biological factors include chemical and physical contaminants, such as metals, veterinary drugs, pesticides, glass fragments, insects, and many others [7]. In general, diseases caused by microbial pathogens are associated with enteric syndromes, but they are not limited to them, and may affect other organs such as kidneys, liver, and the central nervous system [8]. However, diarrheal diseases affect more than 90% of patients, causing an estimate of 230,000 deaths per year [2,3]. The occurrence of two or more cases of similar illnesses resulting from the ingestion of a common food characterizes a FBD outbreak, except botulism, cholera or chemical food poisoning, in which one case corresponds to an issue due to the severity of the disease [9,10].

FBDs are a major cause of morbidity and mortality worldwide, constituting a growing economic and public health problem [3,11]. The economy is affected due to the costs related to the medical services, the loss of productivity and income of the affected individuals, the closing and losses of businesses resulting from the rejection of the foods involved in the outbreaks and the expenses with investigations [12]. The estimated costs of losses of productivity and medical treatment due to food safety issues in developing countries is estimated to be around $110 billion [13]. In addition, when an outbreak is linked to a restaurant or a product, the commercial value of the brand can be significantly reduced, directly affecting its survival in the market [14]. Many factors contribute to the increase of occurrence of FBDs around the world. For instance, population growth and the growing number of vulnerable persons are key factors. Additionally, the need for large-scale food production, the lack of adequate quality control and inspection by the competent bodies, the disorderly urbanization, the larger supply of food for immediate and collective consumption, the change in eating habits such as increased consumption of fresh vegetables and consumption of street food, the environmental transformations, and the globalization are additional factors that contribute to the rapid dissemination of new pathogens and FBDs [15,16,17].

As a result of the growing urbanization process and the lack of time to prepare meals, eating out of home has become a common reality [3]. The consumption of food, often from restaurants, bakeries, food trucks, bars and cafeterias, makes it hard to determine whether good food-handling practices are adopted in these places [18]. This problem becomes even more pronounced when considering street food, i.e., food traded on stalls and many types of improvised surfaces (folding tables, etc.). Besides being a popular eating habit in many places, particularly in developing countries, street food trade may play an important role for the local economy and also as a tourist attraction [19,20]. In a study conducted in South Africa, almost 80% of the studied street vendors (*n* = 399) were never trained on good manufacturing practices nor on food safety [19]. Food trucks may present problems such as poor basic infrastructure, lack of temperature control of refrigerators and freezers, inadequate hygiene practices on the part of venders, as well as insufficient knowledge about food safety practices [21,22].

Due to the practicality and lower cost of these foods, the habit of eating from these sources has grown. If, on one hand, this type of business has significantly contributed to poverty reduction in many less-developed countries, on the other hand, there are many reports of illnesses resulting from this type of food consumption. The causes are numerous, including precarious infrastructure, lack of hygiene, lack of inspection, lack of training on good handling practices, low quality of raw materials and exposure to insects and dust. For instance, a Brazilian survey with street vendors revealed that only 53% and 23% had satisfactory hand and surface hygiene conditions, respectively [23].

This publication reports results of a survey on the occurrence of FBD outbreaks between 2009 and 2022, in multiple countries, associating them with the source of the implicated foods. The data were taken from publications of competent health surveillance agencies and scientific articles in Pubmed and Webofknowledge databases,, using the following search terms in English and Portuguese: food, food safety, outbreaks, foodborne diseases, eating in, eating out. Our intent was to determine whether there are more outbreaks related to food consumed at home or prepared in commercial establishments, such as restaurants, or even street vendors, to raise awareness about the places that require greater attention by health authorities.

## 2. Countries with the Highest Occurrence of FBD Outbreaks Caused by Food Prepared at Restaurants and Similar Establishments

In the United States, between 2009 and 2015, 5760 outbreaks of FBD were reported, which resulted in 100,939 cases, 5699 hospitalizations and 145 deaths, with an annual average of 800 outbreaks, affecting 15,000 people per year. Due to underreporting, the real numbers are estimated to be at least 9.4 million cases per year, indicating that there is a huge amount of people affected by FBDs that do not enter the official statistics, making monitoring and intervention measures difficult [10]. Norovirus was the major agent of these outbreaks (38%) followed by *Salmonella* (30%). On the other hand, *Listeria monocytogenes* and Shiga toxin producing *Escherichia coli* (STEC) accounted for 82% of hospitalizations and deaths. Regarding the location where the implicated foods were prepared, restaurants had the greatest contribution (61%), while homes were associated with only 12% of the outbreaks (Table 1) [10,24].

In New Zealand, 13697 cases of FBD were reported in 2018, with campylobacteriosis remaining as the most common disease, followed by cryptosporidiosis and giardiasis. Commercial establishments, including restaurants, supermarkets and bakeries were associated with 39.5% of the outbreaks, while households added up to only 4.7% [25].

Australia has had high rates of FBD, with *Campylobacter* spp. as the main agent involved in bacterial gastroenteritis. Between 2001 and 2016, 65 outbreaks of FBD by *Campylobacter* were reported in the country, 41 of which (63%) of food origin (Table 2). Among these, 20 (49%) were related to the preparation of food at restaurants, and only three (7%) to foods prepared at home. The origin of the cases was not identified in more than a quarter of reported outbreaks [26,27,28].

In Denmark, 51 foodborne diseases outbreaks were reported in 2019, with a total of 1929 cases. As in the previous year, Norovirus was the most frequent cause (37.3%), which affected 932 people. Interestingly, one of the outbreaks was associated with the consumption of Danish raw oysters served at a private party. There was an increase in the number of outbreaks by *Clostridium perfringens*, which was the second most frequent causal agent (19.6%), and the largest outbreak, involving 268 people, was caused by insufficient cooling of meat sauce. The most frequent location of these outbreaks was “restaurants” (29%) with 15 outbreaks affecting 534 people (average of 38 people per outbreak). Outbreaks in school or work canteens also had a strong impact, affecting 723 people (average 72 people per outbreak), with Norovirus being the main causal agent [29].

In India, between 2009 and 2018, 2688 outbreaks of FBD were reported, resulting in 153,745 cases and 572 deaths. This represents an annual average of 269 outbreaks, 15,375 patients and 57 deaths (2.2 outbreaks per 10 million individuals, with a maximum of 3.2 in 2016) [30]. Most diseases were of bacterial origin, caused mainly by *Staphylococcus aureus*, *Bacillus cereus*, *Escherichia coli*, *Salmonella* and *Vibrio parahaemolyticus*. The majority of outbreaks reported in India affected more than 30 persons per outbreak, mainly in public gatherings such as temples, weddings, canteens, school lunches and community festival celebrations. Those outbreaks with less than 30 cases per outbreak occurred more frequently in a domestic environment [30].

In some countries belonging to the Eastern Mediterranean region according to the World Health Organization (WHO), the majority of FBD outbreaks reported in the last years occurred outside home. In Bahrain, *Salmonella* was the main pathogen involved in the last outbreaks in restaurants. In Egypt, in 2013, a FBD outbreak affected 500 students that consumed bad tuna. However, the notifications were mainly linked to tourists, with hepatitis A virus and *Salmonella* found in the investigated foods consumed in cruise ships. Iran had 2250 notified outbreaks between 2006 and 2011, according to Centers for Communicable Disease Control. The most common pathogens were *E. coli*, *Shigella*, hepatitis A virus, and *Vibrio cholerae*. Foodborne botulism was identified in a survey that evaluated 2037 suspected cases between 2007 to 2017, where 12.3% of these cases were positive, and the sources of the botulism toxins were processed fish, commercially canned goods, and non-pasteurized dairy products [13].

In Canada, *Campylobacter* is the leading cause of enteric diseases, followed by *Salmonella*. The main sources of *Campylobacter* are chicken breasts, broiler chicken, swine, turkey and feedlot beef manure and this illness is associated with improper handling and consumption of raw meat [31]. The primary subtype found in human cases was *Campylobacter jejuni* and a seasonal trend was observed: the cases prevailed in late summer and early fall [32]. The serotypes of *Salmonella* most commonly associated with human diseases in that country are Enteritidis, Typhimurium and Heidelberg. The incidence of non-typhoid salmonellosis increased 10% between 2003 and 2009, reaching 21 cases per 100,000 people in 2016, following a seasonal pattern [33,34] and the majority of foodborne outbreaks occurred outside home, either in the community or in a food service establishment, corresponding to 74% of cases between 2008 and 2014 [35].

In Malaysia, between 2013 and 2018, 21 annual foodborne outbreaks were reported, where *Bacillus cereus* and *Staphylococcus aureus* were the main causal agents, responsible for eight and six outbreaks, respectively, followed by *Salmonella* and *Vibrio parahaemolyticus*, responsible for five and two outbreaks, respectively. Public schools with kitchen, followed by school canteens were the most common places responsible for the outbreaks, usually involving cross contamination. High temperatures and the humid climate that are characteristic of the country favor microbial growth and the inadequate control of temperature and storage conditions during preparation of foods contributed as well [36,37].

## 3. Countries with the Highest Occurrence of FBD Outbreaks Caused by Food Prepared at Home

In China, between 2003 and 2017, 19,517 outbreaks of FBDs were reported, resulting in 235,754 patients and 1457 deaths. There has been an increase in the number of outbreaks over the years, especially since 2011, which led to an annual estimate of 2243 outbreaks, 21,509 patients and 131 deaths. The proportion of outbreaks per million increased from 0.6 in 2003 to 3.9 in 2017. The main causes of outbreaks were poisonous mushrooms (32%), *Vibrio parahaemolyticus* (11%), *Salmonella* (7%), *Staphylococcus aureus* (4%), *Bacillus cereus* (3%), *Proteusbacillus vulgaris* (2%) and *E. coli* (2%). 46.6% outbreaks took place at home, 22.5% at restaurants, 18.4% in canteens and 3.8% in street stalls. Despite the household being the place of highest occurrence, most of these outbreaks resulted from the ingestion of poisonous mushrooms and plant toxins [38]. 

Data from the latest report by the European Food Safety Authority (EFSA) and the European Center for Disease Control and Prevention (ECDC) highlights the social impact of FBDs. There have been 5175 outbreaks reported in 2019 among the 27 EU State members, 49463 cases, 3859 hospitalizations, resulting in 60 deaths. This represents a 50% increase over the previous year, with many of these deaths at institutions for vulnerable populations [39]. Most fatal cases were caused by *Listeria monocytogenes*. *Salmonella* was the most-detected pathogen, but there was an important increase in outbreaks associated with Norovirus due to fish consumption. The highest prevalence of outbreaks was at home (41.3%), while restaurants, cafes, bars, and street food accounted for 28.6% of the outbreaks (Table 3). It was noted that while at home there is a predominance of *Salmonella* and Norovirus, in other places other pathogens are also encountered [39].

**Figure 1:**
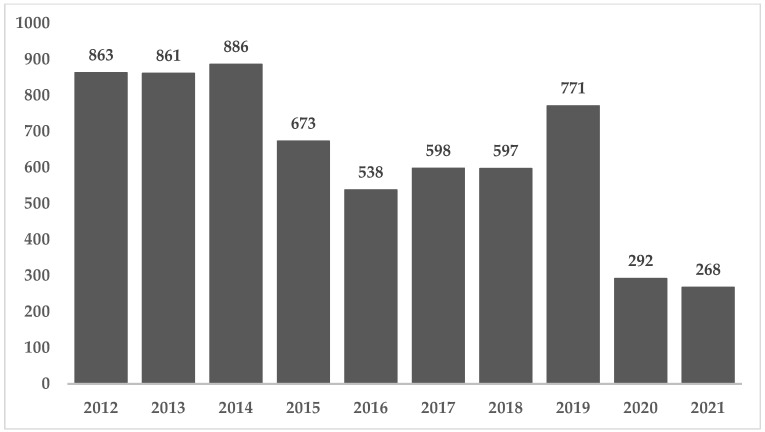
Numbers of FBD outbreaks in Brazil between 2012 and 2021. Adapted from [8].

**Figure 2:**
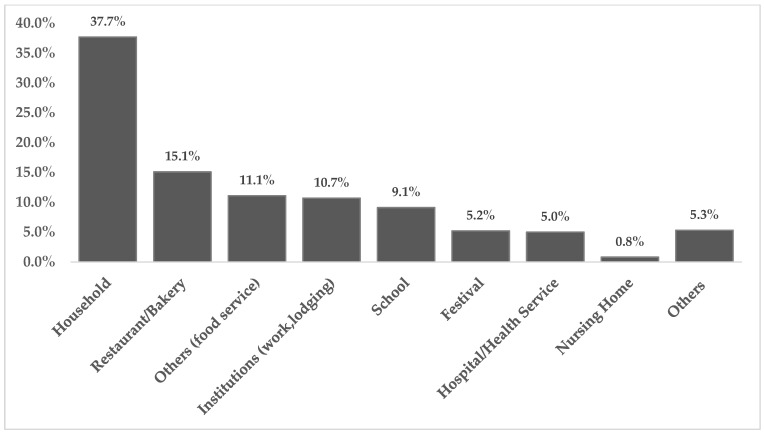
Percentage of FBD outbreaks by place of occurrence in Brazil between 2012 and 2021 Source: Adapted from [8].

In Brazil, between 2009 and 2018, 6809 outbreaks were reported, which resulted in 120,584 patients and 99 deaths. The annual average was 681 outbreaks and 12,058 patients, with a downward trend from 2015 onwards (Figure 1). Households have usually been the main place of occurrence associated with FBD outbreaks, representing 35.8% in 2018 (Figure 2) [16]). In 2017, the main pathogens identified as solely responsible for the outbreaks were *E. coli* (46%), followed by *Salmonella* spp. (15%). Between 2012 and 2021, the pathogens responsible for outbreaks were *E. coli* (29,6%), *Staphylococcus aureus* (12,9%), *Salmonella* spp. (11,2%) and *Bacillus cereus* (7,2%) and water remained the main transmission vehicle, followed by mixed foods [8]. It is important to note that even though FBD outbreaks in Brazil are underreported, the numbers highlight the need of public health interventions aiming to improve food safety practices either at home or at commercial food establishments [16]. The COVID-19 pandemic has affected health surveillance agencies in Brazil, which directed most of their efforts to control the spread of SARS-CoV-2. The downward trend observed in 2020 and 2021 can be explained by the larger number of people working from home. A similar phenomenon occurred in US, where the incidence of infections by foodborne pathogens decreased 26% compared to 2019 [40].

## 4. Discussion

Important actions for harmonizing sanitary measures between countries were established. In the 1990s, among the agreements signed by the World Trade Organization (WTO), the Sanitary and Phytosanitary Agreement (SPS) determines that members must follow international sanitary standards or recommendations, and be part of international organizations and subsidiaries, particularly the Codex Alimentarius Commission (CAC). Good Agricultural, Manufacturing and Handling Practices, as well as Hazard Analysis and Critical Control Points (HACCP) are very important and disseminated tools that help to minimize, eliminate, or reduce contamination by pathogens along the food production chain [41].

Despite these efforts for the international standardization on food safety protocols, countries have different levels of controls in their domestic food chain, particularly because of technological differences, food production traditions, cultures, and topographical and climatic conditions. While developed countries are successful in monitoring their food systems, many developing countries face enormous challenges in the food supply chain [42]. In addition to all the implications for food safety, there is another important factor that must be considered when assessing the existence of foodborne outbreaks, which is the existence of a large number of unreported cases. As an example, it is estimated that in the US, in the National Outbreak Reporting System (NORS), only 0.03% of outbreak cases and 0.7% of hospitalizations caused by foodborne illness were actually reported between 2009 and 2016, which may limit the actions of regulatory and inspection bodies [14,43]. This discrepancy may be even more pronounced in developing countries, including the Latin American and Caribbean region, in which health surveillance agencies may consider FBD as less urgent issues [44].

There are several factors that lead to food contamination, including lack of hygiene with utensils and equipment leading to cross contamination, poor origin of raw materials, infected people in production and/or distribution and inadequate handling or storage. The factors that support the proliferation of pathogens are thawing out of refrigeration, prolonged exposure to room temperature and preparation of large quantities and/or with excessive anticipation [45]. In Brazil and in many other countries, such as the United States and China, inappropriate handling is identified as one of the main causes of FBD outbreaks in commercial, institutional food services and at home [46].

Concerning commercial and institutional establishments, many training programs on good handling practices, based on robust regulations, have not been sufficient to contain the increase of FBD outbreaks in most countries. This is because most interventions exclusively aim at increasing knowledge about good handling practices, which, although essential, do not ensure attitudinal change. A systematic review showed that only 3 out of 23 interventions in commercial and institutional food services were based on the Theory of Planned Behavior or the Rational Action Theory, approaches which link education with action [47]. This discrepancy between episteme and praxis is associated with the multidimensional character of food production environments, in which the values and beliefs of leaders and peers, the administration system, the leadership style, the communication approach and the environment as a whole are determinant in the engagement and the perception of the chance for an FBC case to occur [46].

A survey carried out with 32 food services, many of which serve more than a thousand meals a day in Brazil, showed that the better the leadership and the higher the level of knowledge, the lower the chance of FBD occurrence, which highlights the importance of leaders in promoting an environment capable of motivating organizational commitment and adherence to good hygiene practices [46]. Regarding households, it was found that inadequate practices result from the deficit of information about efficient and safe acquisition, preparation, cooking and food storage, as well as the lack of a clean environment and personal hygiene. There is often a misperception regarding microbiological hazards, and consequently negligence during home preparation and storage including thawing frozen food outside the refrigerator, cross contamination and reheating cooked food under insufficient time and temperature. The chances are higher for younger individuals (less than 20 years old) due to insufficient level of knowledge about food safety and inappropriate practices, elderly people, due to the misguided habits practiced throughout life, and people with a lower level of education [48,49].

Another key issue, especially in less developed countries, is deficient sanitation [45]. According to the World Health Organization, 80% of the diseases in developing countries are spread by water, including FBD, diarrhea, malaria, viral hepatitis, dengue, yellow fever, and other diseases related to vectors that depend on water for their proliferation. Access to safe water is one of the most effective strategies to improve the population’s health and to reduce poverty. Investments in water and sanitation could impact around 9.1% of the Global Burden of Disease, that is, the burden of premature mortality and years lost due to diseases [50,51]. However, data from 2017 showed that globally, approximately three billion people (40% of the population) did not have access to a basic hand washing facility at home, an essential measure to avoid food contamination, more than two billion people still depend on unsafe drinking water supplies and about 673 million people practice open defecation. UNICEF estimates 829,000 diarrheal deaths each year caused by unsafe drinking-water and poor sanitation and hand hygiene [52].

In Brazil, almost 35 million people do not have a treated water supply; the North of the country presents the worst scenario, where only 57.5% of the population is supplied with treated water [11]. Information on water supply can be obtained from SISAGUA (Information System of Water Quality for Human Consumption Surveillance), SNIS (National Sanitation Information System) and the PNAD (National Household Sample Survey), and the surveys are carried out by IBGE (Brazilian Institute of Geography and Statistics). SNIS and SISAGUA aim is to determine the quality of water supply, and through surveillance programs, to implement actions that ensure access to water in accordance with drinking standards, helping to manage health risks. It is important to emphasize that the water quality parameters include only free residual chlorine, turbidity, and counts of fecal coliforms [53,54,55].

It should also be considered that populations with lower purchasing power are the most vulnerable to FBDs, not only because of poor access to safe water, but also because of the need to seek cheaper products in places with less hygienic-sanitary and conservation conditions, and when affected by a FBD, most of the time they do not seek medical care, or cannot pay for it, which worsens their health status over time [56].

It is important to note that the statistics presented, in addition to containing the uncertainty of underreporting resulting from the self-limiting profile of the diseases causing most patients with mild symptoms not to seek medical attention, reflect the model of food consumption habits from before the pandemic of coronavirus disease (COVID-19), where eating out was increasingly replacing meals made at home. Nonetheless, the need for social isolation to contain the spread of the new coronavirus-imposed restrictions on daily in-person activities. Thus, food services that aimed at healthy people who work outside the home reduced their production or stopped activity, while hospitals had to produce on a larger scale and with even more attention to hygienic-sanitary measures given the vulnerability of the public. Interestingly, many food establishments migrated to take away and delivery services in order to maintain their economic activity [57]. Given the restrictions to stay at home, those who were not familiar with cooking or who were looking for practicality, avoiding trips to the supermarkets to buy ingredients, started making use of the food delivery services. At least four major delivery apps became very popular in Brazil during the pandemic [58]. The online food delivery service has grown dramatically in recent years, with a global revenue of $107.4 billion in 2019, and an estimated growth to $182, 3 billion in 2024 [59,60].

Considering that the coronavirus may retain viability for hours or days, depending on the surface, that it is eliminated by sanitizing or disinfecting and properly washing hands; that it is sensitive to the cooking temperature of food (around 70 °C); and that it requires a host to multiply, there is no evidence that food is a source of contamination [61,62,63,64]. Nevertheless, when using a delivery service, the consumer should be aware if the establishments where the orders are placed follow good handling practices, observe the hygiene of the delivery person, if the product is delivered with security seals and properly sanitize the hands after receiving and before eating the products [59,60]. The delivery person is also considered a food handler [65], and the boxes used to deliver ready-to-eat foods and the means of transport should follow the recommendations of good food handling practices. According to [65], delivery people do not undergo periodic training in equipment cleaning and handling, and the conditions of these boxes are often precarious.

Given the increase in delivery services, the market of Dark Kitchens, also called Ghost Kitchens or Cloud Kitchens, was also consolidated; these are shared spaces dedicated exclusively to the production of a menu made available via an app and delivered through delivery services. Virtual kitchens have the same operational process (taking orders, preparing, and packing take-out meals), but have the advantage of being able to offer a greater diversity of menus, brands, concepts, and gastronomic experiences, in addition to reduced costs by optimizing facilities and employees [38]. It is worth mentioning that a Colombian delivery startup, which also operates in Brazil, was one of the first to develop its own kitchen, in which the entire operational process is under its responsibility. In addition to the concern regarding the loss of quality resulting from faster production processes, in Brazil there is also the proliferation of Dark Kitchens favored by the ease of registration on digital platforms, which can have unsatisfactory hygiene, ignoring the standards of the health surveillance agencies [66].

While there was a massive reduction in foodborne infections in the early months of the pandemic, compared to the same period in previous years in the United Kingdom, United States of America, and similar trends in Ireland, Finland and Australia, it is worth noting that these data refer to developed countries, particularly in the initial months of the pandemic, and despite other priorities, there was a reduction in laboratory tests and notifications for other diseases, including FBD, especially for mild cases of gastroenteritis. In any case, the COVID-19 pandemic has given rise to new behaviors in the food sector and from consumers in relation to hygiene practices (especially frequent hand hygiene). In fact, the pandemic has influenced food safety by potentially improving employees’ perception of hygiene, in addition to an increased demand for safe food by consumers, which can positively impact in the control of FBD overall [16,67,68].

## 5. Perspectives and Conclusions

Knowing the sources of foodborne diseases (FBD) is very important for targeted public policies aiming at reducing the burden of FBD. It must be stressed that the detection and recording of FBDs is a global challenge due to the underreporting caused mainly by the self-limiting profile of most of these diseases. Despite this, an increased body of data show that outbreaks are increasing in both developed and developing countries, requiring more effective countermeasures in view of their social and economic impact [10,38].

Regarding the distribution of outbreaks depending on the place where the food was prepared, it was possible to note that there is considerable heterogeneity among countries, making it hard to determine the safety of foods made at home versus those made by street vendors or at restaurants and the like. The question is hard to answer because it involves several cultural factors, such as: eating at festivals and street stalls; eating more frequently at institutional and commercial establishments or at home; consuming raw milk, fish and/or raw vegetables; hygiene habits; demographic factors related to the population’s growth and the growth of the vulnerable groups; political factors related to regulations and surveillance controls by competent bodies in each country; and socioeconomic factors, capable of influencing the quality of water and food.

In Brazil, the data show a higher number of FBD outbreaks associated with home cooking, suggesting that home-made food may be less safe. However, considering that the outbreaks portray only a small portion of the FBD, it would not be appropriate to generalize the data to a conclusion of the safest exposure location. It is also important to consider that the number of residences is much larger than the number of food establishments. Thus, one may conclude that it is actually riskier to eat out (or have the food delivered) than to prepare food at home. Additionally, a large part of the population does not have access to treated and safe water, increasing the chance of microbiological contamination through water and food, especially in households [11].

The economic crisis imposed by the pandemic tends to increase social inequalities [57]. In addition, changes in eating behavior resulting from social isolation, such as more people with less knowledge about good cooking practices at home, greater use of the delivery services and the rise of Dark Kitchens, increase the chance of microbiological contamination overall [66]. The collection of data related to FBD is quite complex, mainly due to underreporting. Even so, it was possible to perceive that less developed countries tend to have worse levels of basic sanitation, and this has a strong impact on the increase in disease outbreaks, including FBDs. A country’s lower level of development also favors the increase of street food vendors as an income alternative, but it is usually associated with a lower level of knowledge on good handling practices, leading to an increase in FBD cases. In order to guarantee the sanitary quality of raw materials and final products offered to the population, the work of regulatory and inspection bodies is crucial and should be broadened, and investments in sanitation should be prioritized in accordance with the 2030 United Nations agenda for sustainable development goals (SGD), particularly SGD-6 [69]. In this context, governments, universities, industries, and other organizations must provide programs to disseminate knowledge on good manufacturing practices to the general public, exploring the available modern communication tools.

## Figures and Tables

**Table 1:** Outbreaks of FBD by location of food preparation in the US and Puerto Rico, between 2009 and 2015.

Location	Outbreaks		Illnesses		Average Number of Cases per Outbreak
	No	%	No.	%	
Restaurants	2880	61	33,465	43	12
À la carte	2239	48	25,150	33	11
Fast-food	369	8	4414	6	12
Self-service	9	0.3	97	0.03	11
Other or unknown	229	5	3231	4	14
Household	561	12	8080	10	14
Multiple system	34	1	573	1	17

Source: [10].

**Table 2:** Characteristics of *Campylobacter* outbreaks by location in Australia, between 2001 and 2016.

Outbreak Site	Foodborne Disease Outbreaks				
	No Outbreak (All Routes)	% Outbreak (% of Food Origin)	Number of Cases (%Laboratory Confirmed)	Median Number of Cases (Min to Max)	Number of Hospitalizations (Number of Deaths)
Restaurants	20	20 (100)	228 (28.90)	10 (2–35)	13
Elderly care	22	8 (36.4)	143 (28)	14.5 (3–49)	10 (2)
Other settings *	5	5 (100)	85 (32.9)	5 (2–63)	1
Camping	7	3 (42.9)	43 (14)	14 (6–23)	0
School	2	2 (100)	42 (38.1)	21 (6–36)	1
Household	9	3 (33.3)	16 (50)	5 (4–7)	0

* Includes three takeout outlets, a sports club, and a dairy farm. Source: [28].

**Table 3:** FBD outbreaks by exposure place in the European Union in 2019.

Type of Settings	No Outbreak	% Of the Total	No. Human Cases	% of the Total	Rate per 100,000 in 2019	Rate per 100,000 in 2010–2018
**Household**	296	41.3	2605	19.0	0.058	0.048
**Institutional canteens or food services**						
School	32	4.5	2407	17.6	0.006	0.009
Residential institution (nursing home or prison or boarding school)	32	4.5	1096	8.0	0.006	0.004
Work	18	2.5	1128	8.2	0.004	0.005
Hospitals or health services	10	1.4	260	1.9	0.002	0.002
Plane, ship, or train	1	0.1	10	0.1	<0.001	0.001
Subtotal	93	13.0	4901	35.8	0.018	0.021
**Restaurants, cafes, bars, street vendors etc.**						
Restaurants, cafes, bars, or hotels	195	27.2	2978	21.8	0.038	0.032
Street vendors	7	1.0	26	0.2	0.001	0.001
Fast-foods	3	0.4	31	0.2	0.001	0.001
Subtotal	205	28.6	3035	22.2	0.040	0.034
**Other places**						
Multiple locations in one country	32	4.5	1214	8.9	0.006	0.001
Camp or picnic	14	2.0	359	2.6	0.003	0.002
Farm	5	0.7	103	0.8	0.001	0.001
Multiple locations in many countries	3	0.4	62	0.5	0.001	<0.001
Fairs and festivals	2	0.3	25	0.2	<0.001	0.002
Others	48	6.7	873	6.4	0.009	0.008
Subtotal	104	14.5	2636	19.3	0.020	0.016
**Unknown**	**18**	**2.5**	**509**	**3.7**	**0.004**	**0.014**
**Total**	**716**	**100**	**13,686**	**100**	**0.141**	**0.133**

Source: [38].
